# In Vitro Cytotoxic Activity of *Coleus hadiensis* Methanolic Extract: Metabolic and Transcriptomic Effects in Lung Cancer Cell Line

**DOI:** 10.3390/molecules31071074

**Published:** 2026-03-25

**Authors:** Ana L. Valdez-Arellanes, Mónica A. Ramírez-Cabrera, Eder U. Arredondo-Espinoza, Emanuel Hernandez-Nuñez, Monica N. Sanchez-González, Isaias Balderas-Renteria, Karla Ramirez-Estrada

**Affiliations:** 1 Laboratorio de Metabolismo Celular, Centro de Laboratorios Especializados (CELAES), Facultad de Ciencias Químicas, Universidad Autónoma de Nuevo León, Ciudad Universitaria, Pedro de Alba S/N, San Nicolás de los Garza 66451, Nuevo León, Mexico; ana.valdezar@uanl.edu.mx (A.L.V.-A.); isaias.balderasrn@uanl.edu.mx (I.B.-R.); 2 Laboratorio de Farmacología Molecular y Modelos Biológicos, Facultad de Ciencias Químicas, Universidad Autónoma de Nuevo León, Vicente Guerrero s/n, Col. Treviño, Monterrey 64570, Nuevo León, Mexico; monica.ramirezcbr@uanl.edu.mx (M.A.R.-C.); eder.arredondosp@uanl.edu.mx (E.U.A.-E.); 3 Departamento de Posgrado e Investigación, Instituto Tecnológico Superior de Calkiní, Av. Ah Canul S/N por Carretera Federal, Calkiní 24930, Campeche, Mexico; ehernandez@itescam.edu.mx; 4 Facultad de Ingeniería Química, Campus de Ciencias Exactas e Ingenierías, Universidad Autónoma de Yucatán, Colonia Chuburná de Hidalgo Inn, Periférico Norte Km. 33.5, Tablaje Catastral 13615, Mérida 97203, Yucatán, Mexico; monica.sanchez@correo.uady.mx

**Keywords:** *Coleus hadiensis*, lung cancer metabolism, cancer metabolic reprograming, plant secondary metabolites, anticancer phytochemicals

## Abstract

Cancer cells resort to metabolic reprogramming to sustain proliferation. Lung cancer has one of the highest mortality rates of all types of cancer. An important factor in its high mortality rate is its tumors’ ability to undergo significant metabolic reprogramming. Phytochemicals can counteract this altered metabolism and exhibit anticancer properties. *Coleus hadiensis*, a plant used in traditional medicine, has shown such potential. This study evaluated the in vitro cytotoxic activity of its methanolic extract and its effects on the metabolism of HTB-177 lung cancer cells. Qualitative and quantitative phytochemical analysis of this extract was performed to characterize its main constituents. Lung cancer cells were treated with different extract concentrations to evaluate their response to the extract. Cytotoxicity was determined using an MTT assay, and metabolites were analyzed through ^1^H-NMR spectroscopy combined with multivariate statistical analysis. Transcriptomic profiling was also conducted to assess gene expression changes in metabolic pathways. Three main phenolic compounds were identified in the extract. The HPLC profile revealed peaks corresponding to gallic acid (GA), ferulic acid (FA), and rosmarinic acid (RA). The extract exhibited cytotoxic activity with an IC_50_ of 192.85 µg/mL. Metabolic alterations were observed mainly in glycolysis, the Krebs cycle, and lipid metabolism—key pathways for tumor growth. Transcriptomic data revealed altered metabolism-related genes. The upregulation of ME1 correlated with the observed increase in pyruvate levels, while the downregulation of ALDH7A1 and ASRGL1 was linked to altered amino acid catabolism. Furthermore, transcriptomic data revealed the upregulation of the pro-apoptotic gene HRK. These results indicate that the methanolic extract of *C. hadiensis* possesses cytotoxic activity against lung cancer cells by modulating central metabolic routes and gene expression linked to cancer cell survival and proliferation.

## 1. Introduction

Lung cancer remains one of the deadliest forms of cancer worldwide due to its aggressive behavior, late-stage detection, and resistance to conventional therapies. Its high incidence and poor prognosis are exacerbated by environmental risk factors such as tobacco smoke, air pollution, and occupational hazards, as well as genetic predispositions [1]. Despite advances in targeted therapies and immunotherapies, survival rates remain low, especially in regions with limited access to specialized treatment, highlighting the urgent need for new therapeutic strategies.

In cancer, a central feature of tumorigenesis is the reprogramming of cellular metabolism [2]. This metabolic shift involves the alteration of key pathways such as glycolysis, glutaminolysis, and lipid metabolism, enabling cancer cells to efficiently generate energy and biosynthetic precursors necessary for rapid growth and division. Unlike normal cells, which primarily rely on oxidative phosphorylation for energy, lung cancer cells often increase aerobic glycolysis (the Warburg effect), allowing them to thrive even in hypoxic tumor microenvironments [2]. In addition, the pentose phosphate pathway becomes highly active, supplying ribose for nucleotide synthesis and NADPH for redox control and anabolic reactions. At the same time, the TCA cycle undergoes functional rewiring, often becoming truncated due to mitochondrial alterations and reactive oxygen species, leading to citrate accumulation. This citrate is then diverted toward de novo lipid synthesis, a pathway markedly upregulated in lung cancer [2].

This metabolic flexibility supports not only proliferation but also resistance to cell death mechanisms and the evasion of immune surveillance, thereby facilitating tumor progression and metastasis [3]. New anti-cancer research suggests that regulating the altered metabolism that benefits the development of these hallmarks is a way of attacking cancer cells. In this context, plant-derived bioactive compounds have garnered significant interest [4].

These natural molecules, terpenes, phenolics, and alkaloids, can disrupt the energy production processes of the cell, such as glycolysis and mitochondrial respiration, or shift the metabolic equilibrium, thereby compromising the cancer cells’ ability to meet their bioenergetic and biosynthetic needs [5].

Medicinal plants are one of the therapeutic alternatives most used by humans, and have been so throughout their evolution, for their preventive and curative qualities against various diseases [6]. Among medicinal plants investigated, species of the *Lamiaceae* family have attracted attention due to their high content of phenolic compounds and terpenoids with reported anticancer activities.

*Coleus hadiensis* is a semi-succulent, herbaceous, highly aromatic plant of the *Lamiaceae* family, native to Africa, where it has been used in traditional medicine for its effects on the digestive and respiratory tracts [7]. This plant is rich in terpenes and phenolic compounds, which exhibit various biological activities, including antibacterial, antifungal, and anti-inflammatory activities [8]. In recent years, the in vitro cytotoxic activity of the plant has been studied in some types of cancer. Previous studies reported anti-proliferative effects of *C. hadiensis* extracts in colon cancer cell lines (HCT-15) and in cervical cancer cells (HeLa) [9,10]. However, its potential against lung cancer remains largely unexplored. The present study focuses on the in vitro cytotoxic activity of the methanol extract of *C. hadiensis* on the lung cancer cell line HTB-177. To better understand the chemical basis of this activity, qualitative and quantitative phytochemical analysis of the methanolic extract was performed to characterize its major constituents.

To further elucidate the biological effects of the extract, we integrated Nuclear Magnetic Resonance (NMR) metabolomics with transcriptomic profiling. This multi-omic approach allows us to reveal the biochemical mechanisms and gene expression changes through which these phytochemicals perturb the metabolic networks sustaining tumor growth [11]. Therefore, the aim of this study was to evaluate the cytotoxic potential of the methanolic extract of *C. hadiensis* on HTB-177 lung cancer cells and to obtain a complete and integrated view of the changes in the cellular metabolome and transcriptome.

## 2. Results and Discussion

### 2.1. Phytochemical Screening

Methanolic extraction of *C. hadiensis* yielded 2.76 g of dry extract, corresponding to a percentage yield of 6.71%. The curative properties of medicinal plants are often attributed to the diverse array of secondary metabolites they produce, such as alkaloids, phenols, saponins, sterols, and more. Phytochemical screening of the methanolic extract of *C. hadiensis* revealed the presence of several key secondary metabolites, including sterols, terpenoids, alkaloids, tannins, saponins, and phenols (Table 1). These findings are consistent with previously reported phytochemical profiles for this plant, validating our preliminary screening results. The detection of these bioactive compounds is crucial, as it forms the chemical basis for the potential therapeutic applications of the plant, potentially leading to future drug discovery and development.

Further quantitative analysis determined the total phenolic content (TPC) of the methanolic extract to be 127.8 mg GAE/g sample. Polyphenols are characterized by the presence of multiple phenol groups and are widely recognized for their antioxidant and other beneficial biological activities, including anticancer properties. The TPC observed in the *C. hadiensis* extract suggests a significant contribution to its bioactivity.

To further elucidate the specific phenolic compounds present in the extract, HPLC analysis was performed. A stock solution of the methanolic extract was prepared at a concentration of 140 mg/mL. The chromatographic profile revealed distinct peaks corresponding to gallic acid (GA), ferulic acid (FA), and rosmarinic acid (RA), which were identified by comparison with commercial standards analyzed under identical chromatographic conditions (Table 2). For their identification, we used a wavelength of 320 nm for rosmarinic acid and ferulic acid and 286 nm for gallic acid.

The quantification of these phenolic acids in the methanolic extract yielded the following concentrations: rosmarinic acid was the highest at 9.73 mg/g DW, followed by ferulic acid at 5.56 mg/g DW, and gallic acid at 0.21 mg/g DW. These three are well-known phenolic acids with documented biological activities, including strong antioxidant, anti-inflammatory, and cytotoxic properties. The RA value was notably high when compared to published data on other *Lamiaceae* species, a family widely known for its RA content. For instance, a comparative study by Benedec et al. [14] using ethanolic extracts reported that *O. vulgare* had the highest concentration (12.40 mg/g), followed by *M. officinalis* (7.84 mg/g), *O. basilicum* (3.59 mg/g), and *R. officinalis* (1.33 mg/g). Our concentration of 9.73 mg/g is superior to that of most of these species and is comparable to the richest sources in the study, suggesting that *C. hadiensis* is an excellent source of RA. Furthermore, the quantification of FA and GA in the *C. hadiensis* extract reveals high concentrations compared to related species such as *C. forskohlii* and *P. barbatus, as* reported in the comparative study by Ganash et al. [15]. Specifically, the ferulic acid concentration was approximately 200 times greater, and the gallic acid concentration was about eight times greater in *C. hadiensis* (0.02755 mg/g and 0.02542 mg/g in *C. forskohlii*, respectively). The identification and quantification of these specific compounds through HPLC provide strong evidence for the observed phytochemical composition and support the traditional uses and potential therapeutic applications of *C. hadiensis*.

### 2.2. Cytotoxic Effect of C. Hadiensis Methanolic Extract

**Figure 1:**
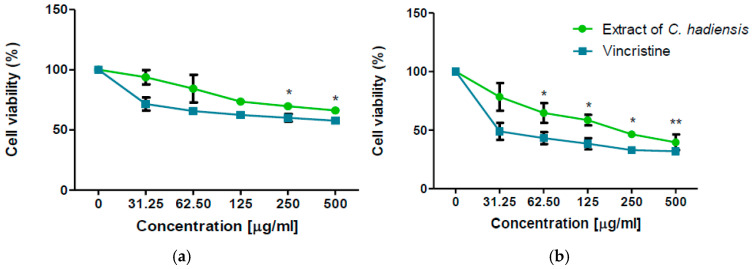
Percentage of cell viability. Vero cells (**a**) and HTB 177 lung cancer line (**b**) after 48 h of exposure to *C. hadiensis* methanolic extract and the anticancer agent vincristine as the reference drug. Test in triplicate. Results are presented as mean ± SD (standard deviation) from three independent experiments. * *p* < 0.05, ** *p* < 0.01, compared to the negative control.

To evaluate the in vitro cytotoxic effect of *C. hadiensis* methanolic extract towards Vero cells and HTB-177 lung cancer cells, dose–response curves were constructed using an MTT assay after 48 h of exposure to the extract (0–500 μg/mL) and to vincristine (for comparison) at the same concentrations. The viability of Vero cells exposed to the *C. hadiensis* methanolic extract decreased in a dose-dependent manner (Figure 1a). At the lowest concentration of 31.25 µg/mL, cell viability was not significantly altered. However, at the highest concentration evaluated (500 μg/mL), we observed a decrease in cell viability to 66%. On the other hand, cells exposed to the lowest concentration of Vincristine decreased in cell viability to 71%, and at the highest concentration to 57%. The IC_50_ values for the methanolic extract and Vincristine in Vero cells at 48 h were 618 and 502 µg/mL, respectively. Although the maximum concentration tested did not reduce Vero cell viability below 50%, the IC_50_ values estimated through linear regression analysis to characterize the trend of the dose–response curve, obtained from the linear regression equation, suggest that the cytotoxic effect on the non-cancerous Vero line occurs at concentrations significantly higher than those required to affect the HTB-177 cancer line.

Human lung cancer HTB-177 cells were more sensitive to the *C. hadiensis* methanolic extract and Vincristine than the Vero cell line; their viability was significantly decreased at concentrations as low as 31.25 µg/mL (Figure 1b). Viability continued decreasing as the concentration of the extract increased. On average, for each increase in concentration, the viability of cancer cells decreased by 10%, suggesting dose-dependent activity. At the highest concentration applied (500 μg/mL), we observed a decrease in cell viability to 39% for cells exposed to the extract and 32% for Vincristine. The resulting IC_50_, estimated by linear regression equation, for the methanolic extract and Vincristine in HTB-177 cells at 48 h was 192.85 and 42.81 µg/mL, respectively, indicating a higher potency of the reference drug. The *C. hadiensis* extract IC_50_ values found for each cell type were used for subsequent assays.

The biological activity of the methanolic extract of *C. hadiensis* may be due to the high content of phenolic compounds in the plant [8]. These types of compounds are known to present anti-cancer activity [16]. According to a phytochemical study conducted by Ji, H and collaborators, this plant has secondary metabolites such as terpenes, flavonoids, and phenolic acids, which we also identified in the preliminary screening. In the methanolic extract, the HPLC analysis was able to identify significant secondary metabolites, including rosmarinic acid, ferulic acid, and gallic acid. These compounds, especially rosmarinic acid, have been attributed to biological activities such as anticancer activity [8,17]. The presence of these specific phenolic acids, identified through HPLC, further supports the potential cytotoxic properties of our extract.

To measure how selective the extract was, the selectivity index was determined. The higher the SI ratio is, the more effective the extract/compound is toward cancer cells [18]. The selectivity index of the extract towards HTB-177 cancer cells was 3.20. According to Prayong et al., a selectivity index greater than 3 indicates high selectivity [18]. The reference drug vincristine, an anticancer drug used in chemotherapy, was used to determine its cytotoxicity in vitro and was compared with the plant extract. As expected, vincristine presented a higher cytotoxic effect in HTB-177 cancer cells than in Vero cells (*p* = 0.0003). The extract showed a dose-dependent decrease in cell viability in both cell lines and exhibited a favorable selectivity index. This suggests that, although the extract is less potent than vincristine, it displays selective cytotoxic activity toward lung cancer cells. Previously, it was reported that *C. hadiensis* plant extracts presented anti-proliferative activity and low cytotoxicity in non-cancer cells [10]. The results obtained in this study support the potential of *C. hadiensis* as a source of natural compounds with selective anticancer activity. Our results suggest that the phytochemicals present in the extract could represent candidates for further investigation in anticancer drug discovery, possibly involving different mechanisms of action from those of conventional chemotherapeutic agents.

### 2.3. Untargeted Metabolomic Effect of C. hadiensis Methanolic Extract on Cell Line Metabolic Profile

**Figure 2:**
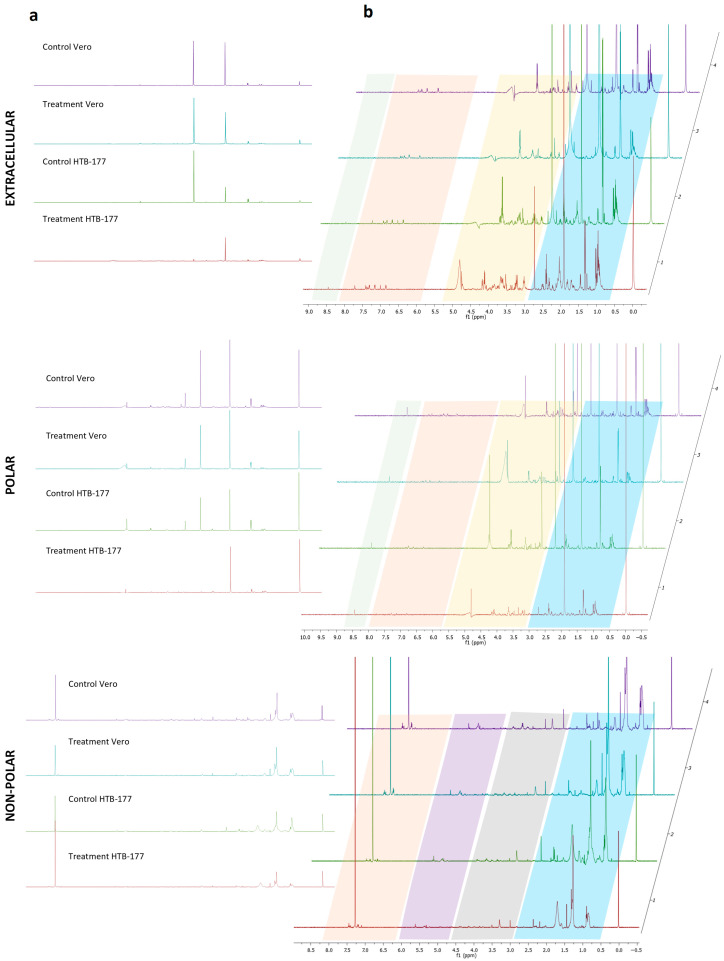
^1^H NMR spectra of extracellular and intracellular (polar and non-polar) extracts of Vero and HTB-177 cells, control and treated. (**a**) is the overview of the spectrum, (**b**) is the close-up. TSP was used as an internal standard at 0.000 ppm for polar extracts and TMS for non-polar extracts. Regions of common signals are highlighted in colors. Blue represents aliphatic area, yellow carbohydrates, orange aromatics, green HCOO^−^, gray glycerin and choline protons, and purple olefinic hydrocarbures.

The representative 1D^1^H NMR spectra of each cell line (treated and untreated) in each of its extracts showed quantitative and qualitative variations. A visual inspection of the spectra revealed differences between the profiles; we observed an increment or decrement in some signal intensities compared to untreated controls (Figure 2). Figure 2a provides an overview of the spectra, revealing the presence of the major signals. For a detailed analysis of the fine structure and discrimination of minority components, a close-up of the regions of interest is presented, allowing a more detailed visualization of the signals (Figure 2b). As is clearly seen, the higher-intensity signals, which are prominent in Figure 2a, exhibit a significant degree of overlap. NMR spectra revealed a reduction in signals in the carbohydrate region (δH 3.0–5.5 ppm) after treatment with the extract, where a greater decrease was observed in the HTB 177 cell samples. The signal intensities in the aliphatic regions (δH 1.0–3.0 ppm), for the polar intracellular, lipophilic, and extracellular extracts decreased, and some signals increased in intensity in both cell lines in response to treatment. In the aromatic region (δH 6.0–8.0 ppm), no marked changes in peak intensities were observed. In the HCOO- region (δH 8.5–9.5 ppm), a signal was found in polar and extracellular extracts that decreased after treatment. Some of the most notable changes were observed in the carbohydrate and aliphatic regions, corresponding to metabolites of glycolysis, the Krebs cycle, and amino acids. These effects suggest a clear reprogramming or modification effect in the cell’s metabolome [19].

The overall effect of exposure to the methanolic extract of *C. hadiensis* on the different extracts obtained from each treated and untreated cell type was analyzed. For this purpose, an untargeted study of the metabolome data (relative areas of the peaks) recorded in 1H NMR for each group of samples was performed. This analysis consisted of bucketing three replicates of each sample. To visualize the main metabolic differences and grouping the samples, a Principal Component Analysis (PCA) was performed for each type of extract.

**Figure 3:**
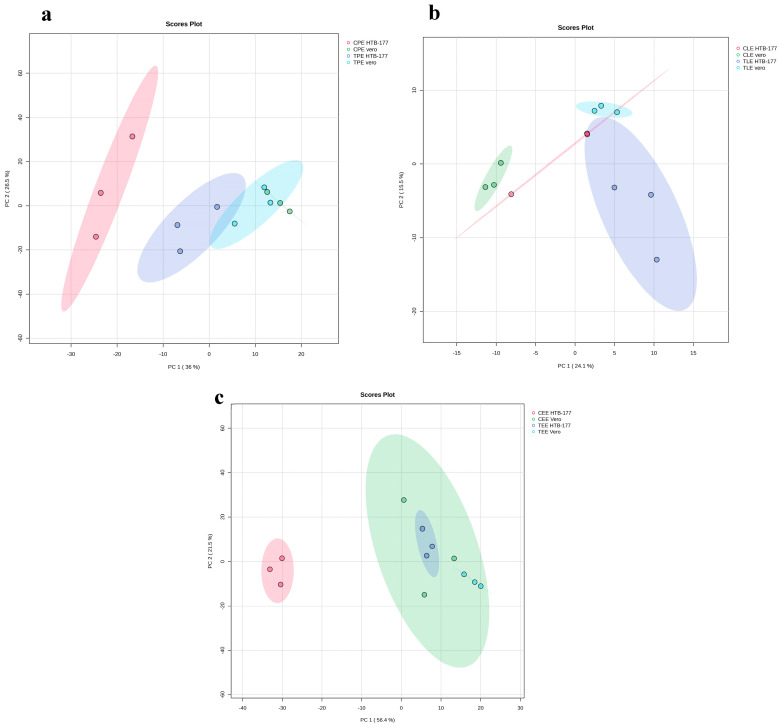
Untargeted PCA. Vero and HTB-177 cell polar extract (**a**), lipophilic extract (**b**), and extracellular extract (**c**) analysis. The green circles are the control Vero cells, light blue is the treatment, the pink circles are the control HTB-177, and the purple circles are the treatment.

In the polar extract (Figure 3a), there is a clear separation between the control HTB-177 cells and the clusters of both control and treated Vero cells. This initial separation suggests that control HTB-177 cancer cells possess a polar metabolite profile that is distinct from that of the non-cancerous Vero cells at baseline. Interestingly, the metabolite profile of the treated HTB-177 cells is then shifted towards the cluster formed by the Vero cell group (control and treated), indicating a metabolic normalization effect induced by the extract. The lipophilic extract (Figure 3b) shows a clear and noticeable separation between the profiles of untreated and treated Vero cells. This indicates that treatment has a significant and consistent impact on the lipophilic profile of Vero cells. HTB-177 cells show a large variability in lipophilic profile due to treatment. The PCA of the extracellular extract (Figure 3c) showed that the untreated HTB-177 cell profile is the most distinct group and is clearly separated from all other groups. This suggests that the extracellular medium, which includes the excreted metabolites, of control HTB-177 cells has a unique and very different profile from that of Vero cells and treated HTB-177 cells. Interestingly, the treated HTB-177 samples were shifted towards the region occupied by both control and treated Vero cells. This indicates that the treatment induces changes in the HTB-177 exometabolome that make it similar to Vero cells. As has been reported in multi-omics studies, the separation between groups in multivariate analyses such as PCA suggests metabolic reprogramming events in response to the extract treatment [20].

### 2.4. Targeted Metabolomics-Metabolic Changes Induced by C. hadiensis Extract

The comparison of the chemical displacements obtained experimentally in NMR and the specific signals reported under similar conditions in Chenomx and in the Human Metabolome Database allowed the identification and relative quantification of 34 compounds (Table 3). Mainly, amino acids and metabolites of the glycolytic pathway, the Krebs cycle, lipids, and ketone bodies were found.

**Figure 4:**
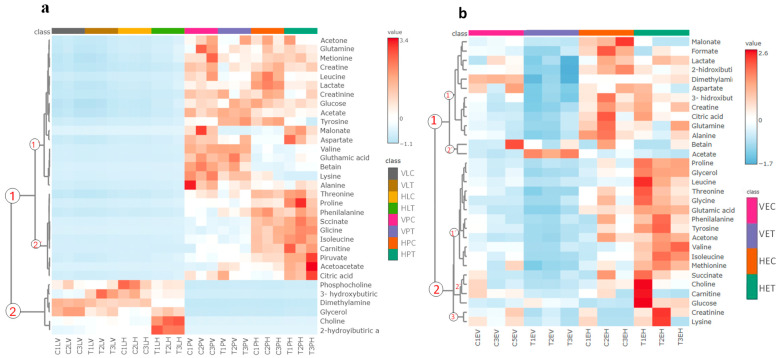
Heatmap of the main metabolite variations from the intracellular (**a**) and extracellular (**b**) metabolic profiles of Vero and HTB-177 cells. Vero extracellular control cells (VEC), Vero cells treated with *C. hadiensis* extract (VET), and the HTB-177 control and treated cells (HEC y HET). Vero lipid extract control (VLC), Vero lipid extract treated (VLT), HTB-177 lipid control (HLC), HTB-177 lipid treated (HLT). Vero polar extract control (VPC), Vero polar extract treated (VPT), HTB-177 polar control (HPC), HTB-177 polar treated (HPT).

To comprehensively visualize the impact of treatment, targeted metabolomic data were organized by hierarchical clustering and visualized as a heatmap (Figure 4). A close examination of this heatmap revealed distinct metabolic profiles across the cell lines and treatments. Two main metabolite clusters were distinguished on the intracellular extract (Figure 4a): cluster (1), corresponding to polar extract, and cluster (2), corresponding to lipophilic extract, each subdivided into two minor subclusters (1’,2’). Analysis of these clusters revealed that cluster (1,1’) mainly contained metabolites that decreased after treatment, including sugars and energy intermediates such as glucose, lactate and acetate, with some amino acids. Cluster (1,2’) contained metabolites involved in the citric acid cycle, such as succinate, citric acid, pyruvate, proline and phenylalanine, which are observed in higher concentrations after treatment in HTB-177 cells. On the other hand, cluster (2) contained lipid-related metabolites such as choline, phosphocholine, and 3-hydroxybutyrate, which were predominant in the lipophilic extracts and were reduced or increased after treatment.

The heatmap of the extracellular medium (Figure 4b) reveals clear metabolic distinctions between cell types and between control and treated conditions. Two major clusters of metabolites were identified (1 and 2), each reflecting coordinated variations in metabolite abundance. The major variations were seen in HTB-177 cells; cluster (1) shows metabolites that decrease after treatment, including glycolysis, the citric acid cycle, and lipid metabolism metabolites such as lactate, formiate, and 2-hidroxibutiric acid. Cluster (2) shows metabolites that increase after treatment, mainly amino acids and glucose. These indicate that the treatment induced coordinated metabolic shifts, favoring the specific pathways of energy, amino acids, and lipid metabolism.

**Figure 5:**
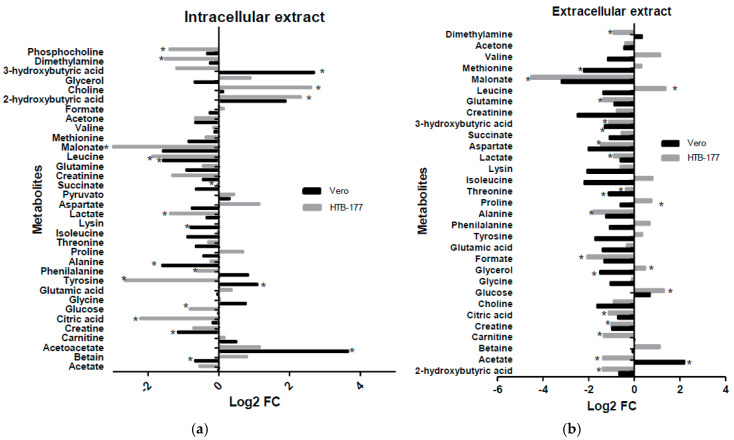
Differential metabolites. Comparison between treated and control samples of the polar and lipophilic intracellular extracts (**a**) and extracellular cell extracts (**b**) of Vero cells and HTB-177. * Indicates metabolites that have Log2(FC) with a *p*-value < 0.05.

To complement the overall visualization of metabolic variations, fold change (FC) analyses expressed on a Log2 scale were performed to quantify the differences in metabolite concentration (Figure 5).

Lipid metabolism: 4 ketone metabolites were found in lung cancer cells, which were 2-hydroxybutyrate, 3-hydroxybutyrate, acetoacetate, and acetone. Elevated levels of all ketone bodies have been reported in patients with lung cancer, as they are common features of cancer metabolism [21]; the increase in these ketone bodies is due to the positively regulated oxidation of fatty acids [22]. These are released into the environment, as they can be used as a source of energy in the absence of glucose in the blood, particularly 3-hydroxybutyrate [23]. These compounds decreased in concentration in HTB-177 cells in both extracts (intracellular and extracellular) after treatment, except for acetoacetate and intracellular 2-hydroxybutyric acid (Figure 5a). The decrease in these metabolites in our study suggests that cancer cells were reprogrammed to normal metabolism after the treatment.

Interestingly, we found that extracellular acetate in HTB-177 cells decreased, while in Vero cells it increased after treatment. Likewise, we observed a decrease in intracellular acetate in the HTB-177 line and no change in Vero (Figure 5b). Acetate is a product of acetyl-CoA; in cancer cells, it supports energy production and lipid synthesis for cell growth. It has been reported that cancer cells excrete acetate into the environment so it can be used by adjacent cells that are poor in nutrients [24]. Our results suggest that *C. hadiensis* extract might decrease the communication by acetate between cancer cells and stop the acetate supply, and thus, the energy for the growth of adjacent cells.

Regarding the metabolites dimethylamine and phosphocholine, there was a decrease in both molecules in both cell lines after treatment, which was statistically significant in the cancer line (Figure 5a). We found a higher concentration of phosphocholine in non-treated HTB-177 cells compared to the Vero cell line. Phosphocholine production occurs through the phosphorylation of choline by the enzyme choline kinase in the first step of the synthesis of phosphatidylcholine, the main phospholipid of the cell membrane. Choline kinase is known to be elevated in highly active tumors due to rapid proliferation [21], as it uses large amounts of choline to produce phosphocholine for the synthesis of membrane phospholipids in demand for cell proliferation. Our results show that after treatment with the *C. hadiensis* extract, phosphocholine concentration decreased, and choline increased in the cancer cell line. This suggests that the plant extract regularized or decreased this phospholipid synthesis pathway, which is necessary for cancer cell proliferation. In addition, a decrease in glycerol in Vero cells and a slight increase in HTB-177 cells were observed after the treatment. Glycerol is part of lipid synthesis, so the extract was able to alter this important part of metabolism, mostly in cancer cells.

Polar metabolism: It was observed that the most concentrated metabolites in the polar fraction of cancer cells were glucose and lactate (derived from glycolysis), succinate, and amino acids such as isoleucine, leucine, phenylalanine, and tyrosine (important in the Krebs cycle). These metabolites decrease after treatment, and other important metabolites of these pathways increase, such as citric acid and pyruvate (Figure 5b). We will explain the relations of these metabolites’ concentration changes in the metabolic pathway that they are part of.

Glycolysis: It is known that lactate concentration is increased in tumoral cells because of the Warburg effect [25]. This effect is defined as an increase in the rate of glucose absorption and preferential lactate production, even in the presence of oxygen, leaving aside the production of energy by the metabolism of pyruvate in the mitochondria. In this study, we observed a significant decrease in intracellular and extracellular lactate production in both cell lines, and a considerable increase in pyruvate production, suggesting an inhibition of lactic fermentation. In addition, we observed a significant decrease in intracellular glucose concentration in HTB-177 cells and an extracellular increase in this metabolite in both cells. The simultaneous increase in pyruvate and decrease in lactate concentration could indicate a redirection of glycolytic output away from lactate production or an increased flux through pyruvate dehydrogenase into the mitochondria. These findings suggest that the extract had a regulating effect on glycolytic activity. As is well-known, cancer cells consume more glucose than normal cells [26]. Our results suggest that the extract managed to inhibit the absorption of glucose and its use through glycolysis. Several studies have found that some natural products can suppress cancer progression by regulating glucose metabolism, including proteins like glycolytic enzymes or glucose transporters [27]. These effects increase the concentration of glucose outside of the cell and affect lactate production. Therefore, both metabolites decrease inside the cell, thus inhibiting the Warburg effect. Previous studies have reported that rosmarinic acid, which, as mentioned before, was one of the secondary metabolites found in the *C. hadiensis* extract, has this anti-Warburg effect [28]; therefore, our results could be due to the possible presence of this metabolite in the extract.

Krebs cycle: Another pathway that is affected and modified in lung cancer is the Krebs cycle, which is downregulated. The characteristic metabolic alteration in tumor cells, known as the Warburg effect (aerobic glycolysis), directly contributes to this change. The Warburg effect shifts the cell’s energy production towards lactate synthesis, decreasing the flow of pyruvate into the mitochondria for complete oxidation in the Krebs cycle. However, cancer cells still require a high volume of fatty acids to divide and replenish their membranes. To satisfy this need, they prioritize the increased intracellular synthesis of citrate. This production often involves the reversal and truncation of the Krebs cycle, diverting citrate away from the energy-producing pathway and channeling it towards lipogenesis [29]. In this study, we observed a high concentration of citric acid in the HTB-177 cell line and a significant decrease after treatment. The reduced levels of cytosolic citrate found in our post-treatment results suggest a limited availability of this metabolite to support lipid synthesis [30] and thus accelerated cell division. This suggests the regulation of lipogenesis and the Krebs cycle, and therefore a decrease in the Warburg effect due to the treatment. This would mean a shift towards a non-cancerous or normal metabolism after the treatment.

Another important metabolite in tumor cells is succinate. This metabolite, which was found in high amounts in the analyzed tumor cells (HTB-177), showed a significant reduction in both intracellular and extracellular extracts after treatment. Succinate has been considered an oncometabolite and a biomarker of cancer, since excess succinate in the cytoplasm of cancer cells is secreted to the extracellular environment, where it promotes cancer cell migration and cancer metastasis [31]. As the Krebs cycle is downregulated, most of the succinate production occurs from glutaminolysis because it comes from α-ketoglutarate. Glutamine and glutamate did not show a significant difference after treatment, so the explanation of why succinate concentration decreased in our study may be due to malonate. Intracellular and extracellular malonate decreased in both cells, which was statistically significant in HTB-177 cells. It has been reported that in cancer cells, there are mutations that cause a low production of succinate dehydrogenase [32], which also explains the accumulation of succinate in the cell. Malonate is a competitive inhibitor of the enzyme succinate dehydrogenase, which produces an accumulation of succinate by inhibiting the transformation of succinate to fumarate [33]. A decrease in the concentration of malonate would partly explain the decrease in succinate.

Amino acid metabolism: A substantial decrease after treatment was found in several amino acids—threonine, isoleucine, lysine, leucine, phenylalanine, and tyrosine—in both cell lines in the intracellular extract, with a significant decrease in the latter three. It has been reported that the levels of these amino acids are increased in lung cancer [34]. This behavior was observed in HTB 177 cells before treatment. It has been documented that there is a downregulation of genes involved in the metabolism of these amino acids within lung cancer tumors. This suggests a decrease in the ability of lung cancer cells to metabolize these amino acids [21]. Our results suggest that cancer cells regain or reactivate the metabolism of these amino acids after treatment.

Exometabolome: In the extracellular extract (corresponding to the culture medium), we found an increase in the concentration of amino acids such as isoleucine, leucine, phenylalanine, tyrosine, proline, and valine (Figure 5b). These metabolites are found in higher concentrations after treatment, which would indicate a lower incorporation of these molecules into cancer cells. LAT1 (amino acid transporter type 1) is one of the most studied amino acid transporters for cancer drug development. Many studies have shown that LAT1 is overexpressed and plays a key role in certain types of cancer [35], such as lung cancer. Inhibition of LAT1 activity leads to an intracellular decrease in amino acids. This would explain the decrease observed in the intracellular extract and, therefore, the increase in its concentration in the extracellular extract.

Other metabolic pathways: Creatine and creatinine have been reported to increase in lung cancer cells, as we observed in our results. Increased creatine levels in tumor tissues lead to an increase in ATP production, which is associated with the highly energetic process of tumor growth and proliferation [36]. Both metabolites come from glycine metabolism; we observed that the concentration of these two molecules decreased in both extracts (intra- and extracellular) in both cell lines. This suggests a broader metabolic modulation by the extract that, in normal cells, may reflect a general shift towards optimized energy expenditure or a non-detrimental alteration in baseline metabolic activity. It has been shown that Vero cells can undergo modifications in metabolic pathways when given external treatment [37]. This suggests that extracts may have broad effects, not necessarily specific to tumor cells. However, in the context of cancer cells, this reduction is particularly significant as it likely disrupts their elevated ATP production, thereby affecting their proliferation. Our results, therefore, suggest that treatment with the extract can alter this process of obtaining energy, affecting the cancer cell and, therefore, decreasing its proliferation.

**Figure 6:**
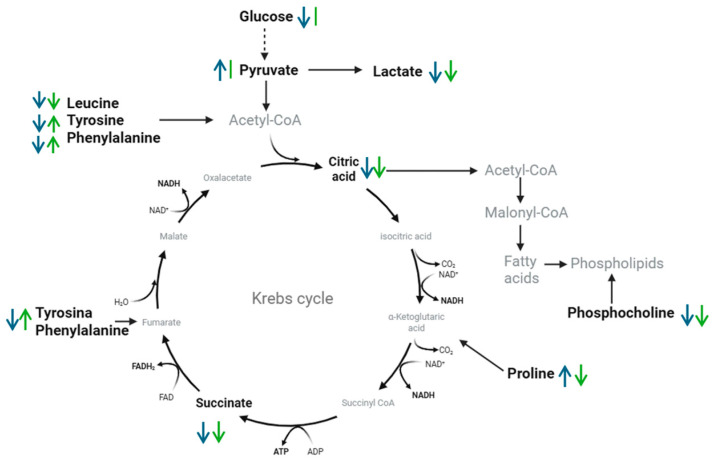
Metabolic map of main pathways affected in HTB-177 and Vero cells. (↑) overproduced, (↓) downproduced, (|) no differences. Blue describes HTB-177, while green describes Vero.

In summary, the observed modulation in the levels of glucose, lactate, pyruvate, citrate, succinate, and several key amino acids suggests a metabolic reorganization in cancer cells, reversing the Warburg effect and limiting the availability of precursors for proliferation. A schematic representation of the main changes detected in these key metabolic pathways in HTB-177 and Vero cells following treatment with the *C. hadiensis* extract is presented in Figure 6.

### 2.5. Transcriptomic Profile

To explore the changes in the transcriptome of Vero and HTB-177 cells after treatment with the methanolic extract, we performed a Microarray analysis. We identified 673 upregulated genes and 500 downregulated genes in treated HTB-177 versus control cells, and 701 upregulated and 477 downregulated genes in treated Vero cells versus control cells.

**Figure 7:**
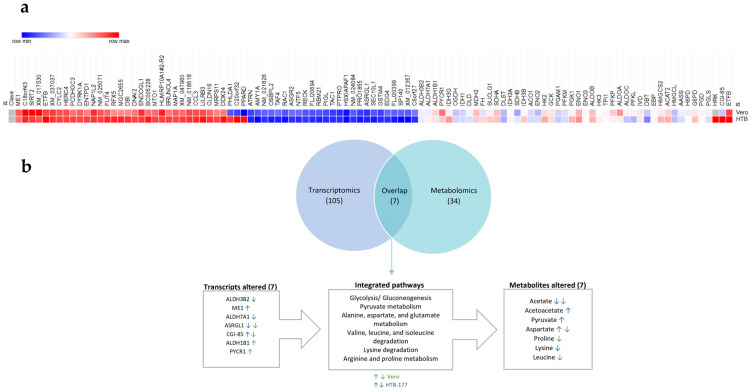
The transcriptome analysis Z score of Vero and HTB-177 cells under the methanolic extract. (**a**) Heatmap, (**b**) Venn diagram of the transcriptomic and metabolomic enriched pathway.

The heatmap shows the top 105 common and significantly expressed genes identified in both cell lines (Figure 7a). To further identify the major molecular pathways and gene functions, the genes were mapped to terms in the KEGG database for gene annotation [38]. The analysis revealed that 359 pathways involved in a wide range of physiological and pathophysiological processes were affected by the methanolic extract. The top 20 changed pathways are in Table 4. Gene functional enrichment analysis was performed using the DAVID tool [39], as well as the identification of significantly enriched pathways based on KEGG. A *p*-value of ≤0.05 was considered statistically significant for enrichment analysis. In an overview analysis, we directly used the FDR values provided by DAVID. More particularly, genes related to glycolysis, the Krebs pathway and amino acid metabolism (lysine), including ME1 and CGI-85, were found. These last two genes, related to lysine metabolism, were significantly upregulated in HTB-177-treated cells, which would explain the decrease in acetate and lysine and the increase in pyruvate after treatment with the extract. The overexpression of genes such as ME1 and CGI-85 in HTB-177 cells suggests an adaptive change that favors the generation of biosynthetic precursors and resistance to oxidative stress. ME1 catalyzes the oxidative decarboxylation of L-malate to pyruvate, producing NADPH. Upregulation of ME1 in HTB-177 and Vero cells treated would directly contribute to an increase in pyruvate levels. ME1’s activity is often linked to anaplerosis (replenishing Krebs cycle intermediates) and the production of NADPH for reductive biosynthesis and antioxidant defense [40,41].

On the other hand, CGI-85, also called KMT5B, a histone lysine N-methyltransferase, catalyzes the dimethylation of lysine 20 of histone H4. The overexpression of KMT5B observed in HTB-177 cells after treatment with the extract could be related to an adaptive epigenetic response. Since KMT5B catalyzes H4K20 dimethylation, its upregulation may reflect an attempt to restore chromatin organization and genomic stability in the face of bioactive compound-induced stress. Previous studies have shown that the increase in KMT5B transcription can decrease the expression of genes (oncogenes) related to proliferation, DNA repair, and cell differentiation [42]. Therefore, the overexpression of the gene in HTB-177 cells suggests an adaptive response to stress and a decrease in tumor proliferation. The decrease in acetate HTB-177 cells (Figure 5a) also suggests a possible detour of this molecule towards histone acetylation pathways or towards acetyl-CoA formation. This assumption is supported by the upregulation of KMT5B, which is an epigenetic regulator. This change reinforces the role of these metabolic and epigenetic pathways in cellular biosynthesis and regulation.

Other genes involved in glycolysis and pyruvate metabolism, like ALDH7A1, and amino acid metabolism (aspartate), like ASRGL1, were downregulated in both cell lines. ALDH7A1 encodes a dehydrogenase involved in the conversion of aldehydes derived from amino acids such as lysine and pyruvate, converting them into their corresponding carboxylic acids to prevent toxic accumulation. Its downregulation may reflect a decrease in the conversion of toxic intermediates and a decrease in mitochondrial oxidative activity [43]. The observed reduction in lysine levels could be due to increased incorporation into proteins or alterations in its catabolism, potentially linked to the downregulated gene ALDH7A1, supporting the idea that the extract modulates anabolic and epigenetic pathways associated with tumor proliferation [44].

In addition, it has been shown that ALDH7A1 influences receptor signaling, such as PPARα, regulating genes involved in lipid metabolism and antioxidant defense. Therefore, the inhibition of its expression (observed in HTB-177 cells) could favor a more glycolytic and less differentiated metabolic profile [45], features commonly associated with tumor aggressiveness. However, this local pro-tumorigenic effect appears to be overridden by the overall action of the *C. hadiensis* extract, which promotes the normalization of key metabolic pathways (Warburg effect reversal) and induces apoptosis. This suggests that the extract’s multi-target synergy prevails over the regulatory role of ALDH7A1 and restores a more oxidative and differentiated metabolic profile, consistent with an antitumor effect.

ASRGL1, responsible for converting asparagine to aspartate, showed reduced expression. Since aspartate is essential in nucleotide synthesis, its decrease may limit cell proliferation or alter energy homeostasis. Studies in cervical cancer and hepatocarcinoma have shown that ASRGL1 diminution induces apoptosis and G2/M phase arrest, reinforcing its role in cell cycle control and tumor differentiation [46,47]. These findings suggest that the plant extract suppresses the expression of genes that could limit proliferation or favor a differentiated metabolism, modulating the metabolism of HTB-177 cells. Although these genes involved in aspartate production were downregulated, aspartate levels were found to be increased in HTB-177 cells after treatment with the methanolic extract. This finding suggests the activation of compensatory metabolic pathways. This type of compensation has been reported in tumor cells, where aspartate is a key metabolite for proliferation, and its availability may determine cell viability against metabolic stress induced by antitumor treatments [48].

Genes involved in other pathways, like HRK, an apoptosis activator, and the ETFB gene, which encodes electron transfer flavoprotein, were significantly upregulated in HTB-177 cells. The HRK gene was found to be overexpressed in lung cancer cell lines after treatment with the extract. Early studies found that low expression of HRK promotes the development and progression of cancer [49]. Therefore, the overexpression found in these cells suggests that *C. hadiensis* extract promotes the apoptosis of lung cancer cells. It has previously been reported that rosmarinic acid, which, as we mentioned before, is present in *C. hadiensis*, positively regulates HRK mRNA expression in triple-negative breast cancer cells. HRK blocks the function of anti-apoptotic proteins and induces intrinsic apoptosis by altering mitochondrial membrane permeability [50]. HRK induction by our extract could represent a promising therapeutic mechanism by restoring intrinsic apoptotic pathways in malignant cells [51].

In Vero cells, genes related to glycolysis and amino acid metabolism were upregulated after treatment, including ALDH1B1 and PYCR1, which would explain the decrease in proline, leucine, and acetate concentration. ALDH1B1, a dehydrogenase located in mitochondria, is involved in the oxidation of aldehydes derived from amino acids such as leucine, as well as in the pyruvate and acetate pathways to acetyl-CoA. Its overexpression could reflect an increased demand for aldehyde detoxification and acetate conversion to lipid synthesis or energy production pathways [52,53]. This activity would also imply an increased flux in the degradation of amino acids such as leucine, which coincides with the decrease in their concentration.

On the other hand, PYCR1 catalyzes the final step in proline biosynthesis from P5C, using NADPH as a cofactor [54]. Its activation is associated with increased cell proliferation and resistance to oxidative stress [55], especially in contexts where regulation of the mitochondrial redox balance is required. The observed decrease in proline in Vero cells after treatment could be due to its accelerated conversion in response to biosynthetic precursor demand [56], especially if the extract promotes anabolic pathways linked to cell growth and antioxidant defense. These findings suggest that the plant extract not only modulates gene expression but also impacts the cellular metabolomic profile, promoting a functional reorganization of energy and amino acid synthesis pathways. This metabolic reprogramming could be directed at maintaining the viability and adaptation of cells to exogenous stimulation [57].

To obtain a comprehensive view of the extract’s impact, pathway analyses were conducted using both transcriptomic data and metabolomic data. The shared findings from these two approaches were identified and visualized using a Venn diagram (Figure 7b). This diagram is a statistical tool that shows the convergent metabolic pathways significantly affected by the treatment, impacting both mRNA expression levels and metabolite concentrations simultaneously. This analysis revealed seven pathways significantly altered by the treatment with the methanolic extract, at both the metabolomic and mRNA expression levels, in Vero and HTB-177 cells. These pathways are involved in glycolysis/gluconeogenesis, pyruvate metabolism, alanine, aspartate, glutamate, arginine, and proline metabolism, and valine, leucine, isoleucine, and lysine degradation. These data indicate that the changes seen in mRNA expression levels may be related to the alteration of some of the metabolic pathways described.

## 3. Materials and Methods

### 3.1. Chemical, Media and Reagents

All reagents and chemicals were purshased form Sigma-Aldrich (St. Louis, MO, USA). HPLC standars were purshased in Cayman Chemical (Ann Arbor, MI, USA). EMEM medium, FBS, Trypsin and TRIzol reagent were purshased in Thermo Fisher Scientific (Waltham, MA, USA).

### 3.2. Plant Extract

*C. hadiensis* plants were grown under controlled conditions from cuttings from an ornamental mother plant, kindly provided by a private donor. The taxonomic identification was previously performed, and a voucher specimen (No. 030597) was deposited in the herbarium of the Facultad de Ciencias Biológicas at the Universidad Autónoma de Nuevo León (UANL). After 5 months of growth, the aerial parts of the plants were collected, freeze-dried and ground. The powdered plant material was macerated at room temperature with 95% methanol, for 48 h. The obtained extract was filtered, the solvent was removed using a rotary evaporator, and the raw extract was stored at −20 °C for further use.

### 3.3. Qualitative Phytochemical Screening

The methanolic extract of *C. hadiensis* was subjected to preliminary phytochemical screening using a concentration of 1 mg/mL to detect the presence or absence of active groups of secondary metabolites utilizing the standard qualitative method of analysis.

Phenolic compounds

Ferric chloride test—Five drops of 10% FeCl_3_ solution were added to the test tube containing 1 mL of plant extract. The appearance of blue-black or green color indicated the presence of phenols in the extract.

Coumarins

For coumarin detection, 1 mg of the sample was reacted with 1 mL of sodium hydroxide (10%). The formation of yellow color in the test sample indicated the presence of coumarins.

Sterols

Salkowski Test—We added 2 mL of chloroform and then carefully added 2 mL of concentrated Sulphuric acid to 2 mL of the plant extract. The formation of a fluorescent greenish-yellow acid layer indicated the presence of sterols.

Saponins

Foam test—One milliliter of the plant extract was diluted with 9 mL of distilled water in a test tube. The mixture was then shaken vigorously for 1 min. The formation of a stable foam layer that persisted for at least 10 min was considered indicative of the presence of saponins.

Terpenoids

In a test tube, 5 mL of plant extract was mixed with 2 mL of chloroform. Subsequently, 3 mL of concentrated sulfuric acid was added to form a second layer. Formation of a reddish-brown precipitate at the interface confirmed the presence of terpenoids.

Alkaloids

Dragendorff’s test—The test was performed by adding 1 mL of Dragendorff’s reagent to 1 mL of the plant extract; the formation of a prominent orange color indicated the test was positive.

### 3.4. Determination of Total Phenolic Content (TPC)

The concentration of total phenolics in the *C. hadiensis* methanolic extract was determined by the Folin–Ciocalteu test. A total of 50 µL of 10 mg/mL methanolic extract and 500 µL of Folin’s phenol reagent (1N) were mixed into test tubes and incubated for 5 min at room temperature (RT). Next, 2.5 mL of 5% sodium carbonate was added, shaken by vortex, and incubated for 40 min at RT. Absorbance was measured at 725 nm. The total phenol content was calculated as gallic acid equivalents from the calibration curve of gallic acid standard solutions (2–13 mg/mL) and expressed as mg of gallic acid equivalent (GAE)/g of extract (on a dry basis). The determination was performed in triplicate.

### 3.5. Identification of Phenolic Compounds by HPLC-DAD

To identify phenolic compounds (PC), the *C. hadiensis* methanolic extract was analyzed by HPLC-DAD in a quaternary pumping system chromatograph equipped with an Agilent Technologies (Santa Clara, CA, USA) 1200 Infinity Series diode array detector. A C-18 chromatographic column (hypersilGold), with a particle size of 5 μm, 250 mm length and 4.6 mm internal diameter was used. The volume of sample injected was 10 μL. The absorbance of the eluted substances was measured at 315 nm.

A binary gradient of phases A and B at a flow rate of 0.8 mL/min was used as the mobile phase. Mobile phase A was composed of water: acetonitrile: formic acid in the ratio 95:5:1 (*v*/*v*/*v*), while phase B was composed of acetonitrile:water:formic acid in the ratio 90:10:1 (*v*/*v*/*v*).

The dry methanolic extract was resuspended in 0.5 mL of HPLC-grade methanol to obtain a final concentration of 140 mg/mL, filtered through a 0.45 μm pore size nylon membrane, and then analyzed by HPLC.

PCs were identified by comparing the retention times and UV spectra of the analyzed samples with commercial standards. For the conversion of area under the curve to concentration terms of the different PC, standard curves were used in a concentration range of 0.01 to 3 mg/mL of the commercial standards. All determinations were performed in triplicate.

### 3.6. Cell Culture

HTB 177 (lung cancer cell line) and Vero cell lines were obtained from the American Type Culture Collection (ATCC) cell bank. Cells were cultured in EMEM medium containing 10% FBS and penicillin/streptomycin (1%) at 37 °C in a 5% CO_2_-humidified atmosphere until a cell monolayer was formed. Both cell lines were maintained as monolayers in 75 cm^2^ plastic flasks. After reaching confluence, the cells were washed twice with 3 mL phosphate-buffered saline (1× PBS) and incubated with 3 mL 0.05% trypsin/0.02% EDTA for 1 min. Trypsin treatment was halted by adding complete media, and the cells were centrifuged (230× *g*, 3 min) and resuspended in complete medium before plating.

### 3.7. Cell Viability Assay

Both cell types (HTB-177 and Vero) were seeded independently in 96-well plates at 1 × 10^4^ cells/ 0.1 mL and allowed to adhere for 24 h. Then, cells were incubated with the previously obtained *C. hadiensis* methanolic extract (31.25, 62.50, 125, 250, 500 µg/mL) for 48 h. Vincristine was used as a comparative control at the same concentrations as the extract. Triton 1% was used as the positive control for cell death, untreated cells were used as a negative control, and a solvent control (DMSO 0.5%) was performed.

Cell viability was estimated by the MTT method. MTT stock solution (100 μL) was added to each well (final concentration 0.5 mg/mL), followed by incubation for 3 h. An isopropyl alcohol: HCL 0.4 N (10:1) solution was added, and the plate was incubated for 30 min in the dark at room temperature. The absorbance was measured at 550 nm. Results were expressed as the percentage of cell viability relative to the control. IC_50_ values were calculated from the concentration–response plot using linear regression analysis with the Graphpad^®^ Prism 7 (GraphPad Software, San Diego, CA, USA). Three independent experiments were performed for each treatment.

### 3.8. Metabolomics

Cell extract preparation. HTB-177 and Vero cells were plated in cell culture flasks (three control samples and three test samples, for each cell line) (~15 × 10^6^ cells/flask) and treated with the IC_50_ of the extract, 192.85 µg/mL for HTB-177 cells and 618 µg/mL for Vero cells. After 48 h of treatment, the medium was recovered from each flask, freeze-dried, and stored for extracellular analysis. The cells were washed once with phosphate-buffered saline (PBS) to remove media components. To extract the intracellular metabolites (hydrophilic and lipophilic), 6 mL of cold methanol was added to each flask, and the cells were scraped from the bottom of the flask. The suspension obtained was transferred to a conical centrifuge tube and vortexed for 30 s. Chloroform (6 mL) and purified water (4.5 mL) were added to each sample tube, followed by 30 s vortex mixing, and the samples were incubated on ice for 10 min. The samples were centrifuged at 4000 RPM for 10 min, and the upper aqueous phase and lower organic phase were then carefully transferred into new Falcon tubes independently. Prior to analysis, the samples were lyophilized and evaporated.

### 3.9. ^1^H NMR Spectroscopy

Lyophilized polar cell extracts and medium were independently resuspended in 500 µL of 0.5 mM TSP in D2O. The organic phase was resuspended in 500 µL of 0.03% TMS in deuterated chloroform. The samples were transferred to NMR tubes.

#### NMR Data Acquisition and Processing

The samples were analyzed on a Varian 600 MHz spectrometer (Palo Alto, CA, USA). The acquisition parameters were as follows: FID size = 64 K, spectral width = 10.50 ppm, gain = 1, acquisition time = 2.18 s, relaxation time = 10 s, and FID resolution = 0.45 Hz. Scan number = 512. 1D ^1^H NMR spectra were processed using Mestrenova v6.0.2-5475 (Mestrelab Research S.L., Santiago de Compostela, España). The free induction decay (FID) signals were multiplied by 0.3 Hz. Spectra were phased, referenced to the TSP or TMS signal at 0.00 ppm, baseline corrected, and the water region and regions devoid of signal at the edges of the spectrum were excluded. The metabolites were identified by chemical shift determination using Chenomx NMR Suite v8.6 (Chenomx Inc., Edmonton, AB, Canada) and consulting the NMR metabolic profiling human metabolome database (HMDB).

### 3.10. Data Analysis

Unsupervised principal component analysis (PCA) was used to show the relationship of the observed variables between experimental treatments and controls. Analyses were performed on a Pareto-scaling data matrix normalized by its sum using the software MetaboAnalyst 4.0. The quality of the models was described by R^2^X and Q^2^ values.

### 3.11. RNA Isolation and Transcriptome Profile

The Vero and HTB-177 cell lines were plated in Petri dishes and conserved at 37 °C in a 5% CO_2_-humidified atmosphere until the formation of a cell monolayer. They were then exposed to the methanolic extract at the IC50 previously obtained. After 48 h, the culture medium was removed. RNA was isolated from the cells using the standard TRIzol protocol. After adding 500 μL of TRIzol reagent to the cell bottle, the mixture was vortexed vigorously. The contents were transferred to Eppendorf tubes. To each tube, 100 μL of chloroform was added and mixed by vortex for 15–30 s. The tubes were incubated at room temperature for 2–3 min and centrifuged at 12× *g* for 15 min at 4 °C. The aqueous phase was transferred to a new tube. Subsequently, 250 μL of isopropyl alcohol was added to every 500 μL of TRIzol used. The tubes were mixed for 10 s and centrifuged at 12× *g* for 10 min at 4 °C. The isopropanol was decanted, and the pellet was washed 3 times with 75% ethanol, vortexed for 10 s and centrifuged at 7.5× *g* for 5 min at 4 °C. This washing was performed three times; in the last wash, the pellet was centrifuged at 12× *g* for 5 min at 4 °C. The pellet was then allowed to dry. Once the pellets were dry, they were resuspended in 30 μL of molecular-grade water, vortexed, and centrifuged for 1 min at 12× *g*. We took 10 μL of the sample and placed it in a new Eppendorf tube for the determination of RNA quality, which was confirmed using electrophoresis and a NanoDrop spectrophotometer (Wilmington, DE, USA). The remaining 20 μL was stored at −20 °C for microarray analysis. Microarray analysis was performed at the DNA Microarray Unit of the Institute of Cellular Physiology, Universidad Nacional Autónoma de México (UNAM). For the analysis, control samples of HTB and Vero were labeled with Alexa555 dye, whereas treated samples were labeled with Alexa647. The results obtained from the image quantification were analyzed using genArise software v1.78.0 (Instituto de Fisiología Celular, UNAM, CDMX, México).

## 4. Conclusions

We detected a difference in the response to treatment with *C. hadiensis* extract between the two cell lines tested. Our data show that the extract affects cancer cells more than healthy cells.

Overall, our results suggest that *C. hadiensis* methanol extract normalizes some points in the metabolic profile of HTB-177 lung cancer cells by modulating key metabolic processes. Alterations of pathways such as glycolysis, the Krebs cycle, and lipid synthesis are known to contribute to the development and progression of lung cancer [2]. As shown in the metabolic map, the effect of the extract is exerted through a synergy of multi-objective effects. The extract acts on several key points of cell metabolism, such as glucose metabolism by counteracting the Warburg effect, protein anabolism by decreasing the level of amino acids, and membrane biosynthesis by reducing phosphocholine. Together, these effects provide a biochemical basis for the cytostatic effect of *C. hadiensis* on HTB-177 cells.

Methanolic extract of *C. hadiensis* could modify the metabolism of lung cancer cells by changing the expression of some genes involved in the metabolism or degradation of metabolites, as well as inhibiting or interfering with transporters and metabolic reactions related to cancer development. At the same time, it suggests that while the extract promotes apoptosis of cancer cells by HRK overexpression, the cells that are still alive change their abnormal metabolism towards a regulated metabolism. All this would imply a decrease in or control over the hallmarks of cancer.

These findings identify the metabolic and transcriptomic basis for metabolism regulation mediated by the methanolic extract of the plant *C. hadiensis* in cancer cells. This leads the way for future research into the multiple mechanisms by which extracts of this plant exert such effects and the exact molecules responsible.

## Figures and Tables

**Table 1:** Phytochemical screening of *C. hadiensis* methanolic extract.

Phytochemical Components	Result	Previously Reported	Reference
Phenols	+	+	[12]
Saponins	+	+	
Alkaloids	+	+	
Sterols	+	+	[13]
Coumarins	+	+	
Terpenoids	+	+	

+ present.

**Table 2:** HPLC-DAD report for phenolic acids detection in *C. hadiensis*.

Compound	t_R_ Standard (min)	t_R_ Extract (min)	λ_max_ (nm)	Concentration (mg/g DW)
Gallic acid	3.61	3.58	286	0.21
Ferulic acid	22.13	22.24	320	5.56
Rosmarinic acid	28.17	28.03	320	9.73

**Table 3:** Altered metabolites and metabolic pathways of Vero and HTB-177 cells influenced by the methanolic extract of *C. hadiensis*.

Metabolic Pathway	Metabolite	Chemical Shift (ppm)	Reference
Glycolysis	Glucose	5.23, 4.64 (3.89–3.23)	HMDB0000122
	Pyruvate	2.4	HMDB0000243
	Lactate	4.1, 1.3	HMDB0000190
Krebs Cycle	Succinate	2.39	HMDB0000254
	Citric acid	2.42, 2.52, 2.66	HMDB0000094
Amino acids	Glutamic acid	2.10, 2.15, 2.36, 3.75	HMDB0000148
	Tyrosine	6.91, 7.21, 3.9	HMDB0000158
	Phenylalanine	3.13, 3.99	HMDB0000159
	Alanine	1.48	HMDB0000161
	Proline	1.99–2.09, 2	HMDB0000162
	Threonine	1.32, 3.58	HMDB0000167
	Isoleucine	1.02, 0.99	HMDB0000172
	Lysin	1.90, 3.77	HMDB0000182
	Aspartate	2.70, 3.89, 2.95, 3.89	HMDB0000191
	Glutamine	2.07–2.17, 2.00–2.10	HMDB0000641
	Leucine	0.96	HMDB0000687
	Methionine	2.11–2.18, 2.63, 3.84	HMDB0000696
	Valine	0.97, 1.04	HMDB0000883
	Glycine	3.56	HMDB0000123
	Betaine	3.27, 3.85	HMDB0000043
	Carnitine	2.4, 3.2, 3.4, 4.6	HMDB0000062
Lipids/phospholipids	Choline	3.55, 4.07	HMDB0000097
	Glycerol	3.43, 3.60, 3.61, 3.85	HMDB0000131
	Phosphocholine	3.21, 3.59, 4.16	PubChem:1014
Ketone bodies	Acetone	2.22	HMDB0001659
	Acetoacetate	2.3, 3.4	HMDB0304256
	2-hydroxybutyric acid	0.89, 1.64, 3.98	HMDB0000008
	3-hydroxybutyric acid	1.19, 2.29, 2.39, 4.14	HMDB0000011
Other	Acetate	1.91	HMDB0000042
	Creatine	3.04, 3.95	HMDB0000064
	Formate	8.4	HMDB0000142
	Creatinine	3.03, 4.04, 4.09	HMDB0000562
	Malonate	3.11	HMDB0000691
	Dimethylamine	2.7	HMDB0000087

**Table 4:** Top 20 enriched KEGG pathways of differentially expressed genes in C. hadiensis-treated cells.

Pathway	Count	*p*-Value	FDR
Metabolic pathways	51	3.27 × 10^−26^	2.3 × 10^−24^
Carbon metabolism	34	3.75 × 10^−47^	5.29 × 10^−45^
Glycolysis/Gluconeogenesis	19	8.25 × 10^−25^	3.88 × 10^−23^
Biosynthesis of amino acids	18	4.8 × 10^−22^	1.69 × 10^−20^
Citrate cycle (TCA cycle)	13	3.45 × 10^−19^	9.72 × 10^−18^
HIF-1 signaling pathway	12	1.91 × 10^−10^	0.000000003
Pentose phosphate pathway	10	3.84 × 10^−13^	9.03 × 10^−12^
Central carbon metabolism in cancer	9	2.65 × 10^−8^	0.000000374
2-Oxocarboxylic acid metabolism	9	4.26 × 10^−11^	8.57 × 10^−10^
Fructose and mannose metabolism	9	7.14 × 10^−11^	1.26 × 10^−9^
Valine, leucine and isoleucine degradation	8	3.33 × 10^−8^	0.000000426
Pyruvate metabolism	7	0.000000768	0.00000902
Galactose metabolism	6	0.00000242	0.0000263
RNA degradation	6	0.000199	0.00175
Lysine degradation	6	0.0000716	0.000721
Glucagon signaling pathway	5	0.00643	0.0432
Tryptophan metabolism	5	0.000195	0.00175
Lipoic acid metabolism	4	0.000336	0.00279
PPAR signaling pathway	4	0.0157	0.0887
Glutathione metabolism	4	0.0079	0.0506

## Data Availability

The datasets generated during and/or analyzed during the current study are available from the corresponding author on reasonable request.

## References

[1] Sung H., Ferlay J., Siegel R.L., Laversanne M., Soerjomataram I., Jemal A., Bray F. (2021). Global Cancer Statistics 2020: GLOBOCAN Estimates of Incidence and Mortality Worldwide for 36 Cancers in 185 Countries. CA Cancer J. Clin..

[2] Chang L., Fang S., Gu W. (2020). The Molecular Mechanism of Metabolic Remodeling in Lung Cancer. J. Cancer.

[3] Bast R.C., Croce C.M., Hait W.N., Hong W.K., Kufe D.W., Piccart-Gebhart M.J., Pollock R.E., Weichselbaum R.R., Wang H., Holland J.F. (2017). Biological Hallmarks of Cancer.

[4] Khan A., Siddiqui S., Husain S., Mazurek S., Askandar Iqbal M., Husain S.A., Iqbal M.A. (2020). Phytochemicals Targeting Metabolic Reprogramming in Cancer: An Assessment of Role, Mechanisms, Pathways and Therapeutic Relevance. Authorea Preprints.

[5] Shuvalov O., Kirdeeva Y., Daks A., Fedorova O., Parfenyev S., Simon H.U., Barlev N.A. (2023). Phytochemicals Target Multiple Metabolic Pathways in Cancer. Antioxidants.

[6] Oteng Mintah S., Asafo-Agyei T., Archer M.-A., Atta-Adjei P., Boamah D., Kumadoh D., Appiah A., Ocloo A., Duah Boakye Y., Agyare C. (2019). Medicinal Plants for Treatment of Prevalent Diseases. Pharmacogn. Med. Plants.

[7] Lukhoba C.W., Simmonds M.S.J., Paton A.J. (2006). *Plectranthus*: A Review of Ethnobotanical Uses. J. Ethnopharmacol..

[8] Ji H.S., Li H., Mo E.J., Kim U.H., Kim Y.H., Park H.Y., Jeong T.S. (2019). Low-Density Lipoprotein-Antioxidant Flavonoids and a Phenolic Ester from *Plectranthus hadiensis* Var. *Tomentosus*. Appl. Biol. Chem..

[9] Menon D.B., Sasikumar J.M., Latha K. (2011). Anti Inflammtory and Cytotoxic Activity of Methanolic Extract of *Plectranthus hadiensis* Stem. Pharmacologyonline.

[10] Menon D.B., Gopalakrishnan V.K. (2015). Terpenoids Isolated from the Shoot of *Plectranthus hadiensis* Induces Apoptosis in Human Colon Cancer Cells via the Mitochondria-Dependent Pathway. Nutr. Cancer.

[11] Chen Y., Gao Y., Yi X., Zhang J., Chen Z., Wu Y. (2020). Integration of Transcriptomics and Metabolomics Reveals the Antitumor Mechanism Underlying Shikonin in Colon Cancer. Front. Earth Sci..

[12] Menon D.B., Sasikumar J.M., Article R., Sasikumar J.M. (2011). Pharmacognostic Study and Phytochemical Investigation of *Plectranthus hadiensis*. Int. J. Pharm. Pharm. Sci..

[13] Ibrahim M.E., Ahmed S.S., Hussein M.S., El-Sawi S.A. (2019). Chemical Investigations and the Antimicrobial Activity of *Ocimum hadiensis* (Forssk) Plant Grown Wild in Egypt. J. Mater. Environ. Sci.

[14] Benedec D., Hanganu D., Oniga I., Brindusa T., Pharm Sci P.J., Tiperciuc B., Olah N.-K., Raita O., Bischin C., Silaghi-Dumitrescu R. (2015). Assessment of Rosmarinic Acid Content in Six Lamiaceae Species Extracts and Their Antioxidant and Antimicrobial Potential. Pak. J. Pharm. Sci..

[15] Ganash M., Qanash S. (2018). Phenolic Acids and Biological Activities of Coleus Forskohlii and Plectranthus Barbatus as Traditional Medicinal Plants. Int. J. Pharmacol..

[16] Nadeem M., Imran M., Gondal T.A., Imran A., Shahbaz M., Amir R.M., Sajid M.W., Qaisrani T.B., Atif M., Hussain G. (2019). Therapeutic Potential of Rosmarinic Acid: A Comprehensive Review. Appl. Sci..

[17] Anwar S., Shamsi A., Shahbaaz M., Queen A., Khan P., Hasan G.M., Islam A., Alajmi M.F., Hussain A., Ahmad F. (2020). Rosmarinic Acid Exhibits Anticancer Effects via MARK4 Inhibition. Sci. Rep..

[18] Prayong P., Barusrux S., Weerapreeyakul N. (2008). Cytotoxic Activity Screening of Some Indigenous Thai Plants. Fitoterapia.

[19] Guerra Â.R., Paulino A.F., Castro M.M., Oliveira H., Duarte M.F., Duarte I.F. (2020). Triple Negative Breast Cancer and Breast Epithelial Cells Differentially Reprogram Glucose and Lipid Metabolism upon Treatment with Triterpenic Acids. Biomolecules.

[20] Li S., Wu R., Wang L., Dina Kuo H.C., Sargsyan D., Zheng X., Wang Y., Su X., Kong A.N. (2021). Triterpenoid Ursolic Acid Drives Metabolic Rewiring and Epigenetic Reprogramming in Treatment/Prevention of Human Prostate Cancer. Mol. Carcinog..

[21] Bamji-Stocke S., Van Berkel V., Miller D.M., Frieboes H.B. (2018). A Review of Metabolism-Associated Biomarkers in Lung Cancer Diagnosis and Treatment. Metabolomics.

[22] Hilvo M., De Santiago I., Gopalacharyulu P., Schmitt W.D., Budczies J., Kuhberg M., Dietel M., Aittokallio T., Markowetz F., Denkert C. (2016). Accumulated Metabolites of Hydroxybutyric Acid Serve as Diagnostic and Prognostic Biomarkers of Ovarian High-Grade Serous Carcinomas. Cancer Res..

[23] Mierziak J., Burgberger M., Wojtasik W. (2021). 3-Hydroxybutyrate as a Metabolite and a Signal Molecule Regulating Processes of Living Organisms. Biomolecules.

[24] Bose S., Ramesh V., Locasale J.W. (2019). Acetate Metabolism in Physiology, Cancer, and Beyond. Trends Cell Biol..

[25] Weber G.F. (2016). Time and Circumstances: Cancer Cell Metabolism at Various Stages of Disease Progression. Front. Oncol..

[26] Adekola K., Rosen S.T., Shanmugam M., Lurie R.H. (2012). Glucose Transporters in Cancer Metabolism HHS Public Access. Curr. Opin. Oncol..

[27] Zhang Y., Li Q., Huang Z., Li B., Nice E.C., Huang C., Wei L., Zou B. (2022). Targeting Glucose Metabolism Enzymes in Cancer Treatment: Current and Emerging Strategies. Cancers.

[28] Han S., Yang S., Cai Z., Pan D., Li Z., Huang Z., Zhang P., Zhu H., Lei L., Wang W. (2015). Anti-Warburg Effect of Rosmarinic Acid via MiR-155 in Gastric Cancer Cells. Drug Des. Devel. Ther..

[29] Parkinson E.K., Adamski J., Zahn G., Gaumann A., Flores-Borja F., Ziegler C., Mycielska M.E. (2021). Extracellular Citrate and Metabolic Adaptations of Cancer Cells. Cancer Metastasis Rev..

[30] Moreno P., Jiménez-Jiménez C., Garrido-Rodríguez M., Calderón-Santiago M., Molina S., Lara-Chica M., Priego-Capote F., Salvatierra Á., Muñoz E., Calzado M.A. (2018). Metabolomic Profiling of Human Lung Tumor Tissues—Nucleotide Metabolism as a Candidate for Therapeutic Interventions and Biomarkers. Mol. Oncol..

[31] Casas-Benito A., Martínez-Herrero S., Martínez A. (2023). Succinate-Directed Approaches for Warburg Effect-Targeted Cancer Management, an Alternative to Current Treatments?. Cancers.

[32] Li T., Copeland C., Le A. (2021). Glutamine Metabolism in Cancer. Adv. Exp. Med. Biol..

[33] Yu S.K. (2002). Malonate Metabolism: Biochemistry, Molecular Biology, Physiology, and Industrial Application. J. Biochem. Mol. Biol..

[34] Dols M.C., Domínguez López M., Ramírez Plaza C., Pérez Miranda E., Gil Calle S., Chamorro E.V., Alés Díaz I., Pino A.M., García J.A., Calderón V.G. (2006). Specific Alterations in the Serum Amino Acid Profile of Patients with Lung Cancer and Head and Neck Cancer. Oncologia.

[35] Yothaisong S., Dokduang H., Anzai N., Hayashi K., Namwat N., Yongvanit P., Sangkhamanon S., Jutabha P., Endou H., Loilome W. (2017). Inhibition of L-Type Amino Acid Transporter 1 Activity as a New Therapeutic Target for Cholangiocarcinoma Treatment. Tumour Biol..

[36] Puchades-Carrasco L., Jantus-Lewintre E., Pérez-Rambla C., García-García F., Lucas R., Calabuig S., Blasco A., Dopazo J., Camps C., Pineda-Lucena A. (2016). Serum Metabolomic Profiling Facilitates the Non-Invasive Identification of Metabolic Biomarkers Associated with the Onset and Progression of Non-Small Cell Lung Cancer. Oncotarget.

[37] Yu S., Yan J., Yang Z., Zhao Y., Wang H., Yang X. (2022). Metabolic Responses and Pathway Changes of Vero Cells under High-Vitamin B Medium. Vaccines.

[38] Kanehisa M., Furumichi M., Sato Y., Matsuura Y., Ishiguro-Watanabe M. (2025). KEGG: Biological Systems Database as a Model of the Real World. Nucleic Acids Res..

[39] Sherman B.T., Hao M., Qiu J., Jiao X., Baseler M.W., Lane H.C., Imamichi T., Chang W. (2022). DAVID: A Web Server for Functional Enrichment Analysis and Functional Annotation of Gene Lists (2021 Update). Nucleic Acids Res..

[40] Heart E., Cline G.W., Collis L.P., Pongratz R.L., Gray J.P., Smith P.J.S. (2009). Role for Malic Enzyme, Pyruvate Carboxylation, and Mitochondrial Malate Import in Glucose-Stimulated Insulin Secretion. Am. J. Physiol. Endocrinol. Metab..

[41] Lu Y.X., Ju H.Q., Liu Z.X., Chen D.L., Wang Y., Zhao Q., Wu Q.N., Zeng Z.L., Qiu H.B., Hu P.S. (2018). ME1 Regulates NADPH Homeostasis to Promote Gastric Cancer Growth and Metastasis. Cancer Res..

[42] López V., Tejedor J.R., Carella A., García M.G., Santamarina-Ojeda P., Pérez R.F., Mangas C., Urdinguio R.G., Aranburu A., de la Nava D. (2021). Epigenetic Deregulation of the Histone Methyltransferase KMT5B Contributes to Malignant Transformation in Glioblastoma. Front. Cell Dev. Biol..

[43] Leandro J., Houten S.M. (2019). Saccharopine, a Lysine Degradation Intermediate, Is a Mitochondrial Toxin. J. Cell Biol..

[44] Shen R., Ruan H., Lin S., Liu B., Song H., Li L., Ma T. (2022). Lysine Succinylation, the Metabolic Bridge between Cancer and Immunity. Genes Dis..

[45] Andrejeva D., Kugler J.M., Nguyen H.T., Malmendal A., Holm M.L., Toft B.G., Loya A.C., Cohen S.M. (2018). Metabolic Control of PPAR Activity by Aldehyde Dehydrogenase Regulates Invasive Cell Behavior and Predicts Survival in Hepatocellular and Renal Clear Cell Carcinoma. BMC Cancer.

[46] Lv X.F., Hong H.Q., Liu L., Cui S.H., Ren C.C., Li H.Y., Zhang X.A., Zhang L.D., Wei T.X., Liu J.J. (2018). RNAi-Mediated Downregulation of Asparaginase-like Protein 1 Inhibits Growth and Promotes Apoptosis of Human Cervical Cancer Line SiHa. Mol. Med. Rep..

[47] Wang X., Wang Y., Yang L., Yuan J., Shen W., Zhang W., Wang J., Tao K. (2023). ASRGL1 Downregulation Suppresses Hepatocellular Carcinoma Tumorigenesis in a CDK1-Dependent Manner. Dig. Liver Dis..

[48] Shi J., Wen K., Mui S., Li H., Liao H., He C., Yan Y., Zhou Z., Xiao Z. (2024). Integrated Analysis Reveals an Aspartate Metabolism-Related Gene Signature for Predicting the Overall Survival in Patients with Hepatocellular Carcinoma. Clin. Transl. Oncol..

[49] Nakamura M., Shimada K., Konishi N. (2008). The Role of HRK Gene in Human Cancer. Oncogene.

[50] Messeha S.S., Zarmouh N.O., Asiri A., Soliman K.F.A. (2020). Rosmarinic Acid-Induced Apoptosis and Cell Cycle Arrest in Triple-Negative Breast Cancer Cells. Eur. J. Pharmacol..

[51] Ranneh Y., Bakar M.F.A., Akim A.M., Baharum Z.B., Ellulu M.S., Fadel A. (2023). Induction of Apoptosis and Modulation of Caspase Activity on MCF-7 Human Breast Cancer Cells by Bioactive Fractionated Cocoa Leaf Extract. Asian Pac. J. Cancer Prev..

[52] Marada A., Korupalli C., Zhao W., Xia Y., Gao Z., Chen J., Zhang E. (2025). Multiple Roles of ALDH1 in Health and Disease. Front. Physiol..

[53] Stagos D., Chen Y., Brocker C., Donald E., Jackson B.C., Orlicky D.J., Thompson D.C., Vasiliou V. (2010). Aldehyde Dehydrogenase 1B1: Molecular Cloning and Characterization of a Novel Mitochondrial Acetaldehyde-Metabolizing Enzyme. Drug Metab. Dispos..

[54] Bogner A.N., Stiers K.M., Tanner J.J. (2021). Structure, Biochemistry, and Gene Expression Patterns of the Proline Biosynthetic Enzyme Pyrroline-5-Carboxylate Reductase (PYCR), an Emerging Cancer Therapy Target. Amino Acids.

[55] Kuo M.L., Lee M.B.E., Tang M., Den Besten W., Hu S., Sweredoski M.J., Hess S., Chou C.M., Changou C.A., Su M. (2016). PYCR1 and PYCR2 Interact and Collaborate with RRM2B to Protect Cells from Overt Oxidative Stress. Sci. Rep..

[56] Phang J.M. (2021). Perspectives, Past, Present and Future: The Proline Cycle/Proline-Collagen Regulatory Axis. Amino Acids.

[57] Encarnação C.C., Faria G.M., Franco V.A., Botelho L.G.X., Moraes J.A., Renovato-Martins M. (2024). Interconnections within the Tumor Microenvironment: Extracellular Vesicles as Critical Players of Metabolic Reprogramming in Tumor Cells. J. Cancer Metastasis Treat..

